# Real-Time Web-Based Assessment of Total Population Risk of Future Emergency Department Utilization: Statewide Prospective Active Case Finding Study

**DOI:** 10.2196/ijmr.4022

**Published:** 2015-01-13

**Authors:** Zhongkai Hu, Bo Jin, Andrew Y Shin, Chunqing Zhu, Yifan Zhao, Shiying Hao, Le Zheng, Changlin Fu, Qiaojun Wen, Jun Ji, Zhen Li, Yong Wang, Xiaolin Zheng, Dorothy Dai, Devore S Culver, Shaun T Alfreds, Todd Rogow, Frank Stearns, Karl G Sylvester, Eric Widen, Xuefeng B Ling

**Affiliations:** ^1^College of Computer Science and TechnologyZhejiang UniversityHangzhouChina; ^2^Department of SurgeryStanford UniversityStanford, CAUnited States; ^3^HBI Solutions IncPalo Alto, CAUnited States; ^4^Department of PediatricsStanford UniversityStanford, CAUnited States; ^5^Department of Electrical EngineeringTsinghua UniversityBeijingChina; ^6^College of Information EngineeringQingdao UniversityQingdaoChina; ^7^Institute of Microanalytical SystemZhejiang UniversityHangzhouChina; ^8^Department of StatisticsStanford UniversityStanford, CAUnited States; ^9^Academy of Mathematics and Systems ScienceChinese Academy of SciencesBeijingChina; ^10^HealthInfoNetPortland, MEUnited States

**Keywords:** ED, machine learning, HIE, EMR, modeling

## Abstract

**Background:**

An easily accessible real-time Web-based utility to assess patient risks of future emergency department (ED) visits can help the health care provider guide the allocation of resources to better manage higher-risk patient populations and thereby reduce unnecessary use of EDs.

**Objective:**

Our main objective was to develop a Health Information Exchange-based, next 6-month ED risk surveillance system in the state of Maine.

**Methods:**

Data on electronic medical record (EMR) encounters integrated by HealthInfoNet (HIN), Maine’s Health Information Exchange, were used to develop the Web-based surveillance system for a population ED future 6-month risk prediction. To model, a retrospective cohort of 829,641 patients with comprehensive clinical histories from January 1 to December 31, 2012 was used for training and then tested with a prospective cohort of 875,979 patients from July 1, 2012, to June 30, 2013.

**Results:**

The multivariate statistical analysis identified 101 variables predictive of future defined 6-month risk of ED visit: 4 age groups, history of 8 different encounter types, history of 17 primary and 8 secondary diagnoses, 8 specific chronic diseases, 28 laboratory test results, history of 3 radiographic tests, and history of 25 outpatient prescription medications. The c-statistics for the retrospective and prospective cohorts were 0.739 and 0.732 respectively. Integration of our method into the HIN secure statewide data system in real time prospectively validated its performance. Cluster analysis in both the retrospective and prospective analyses revealed discrete subpopulations of high-risk patients, grouped around multiple “anchoring” demographics and chronic conditions. With the Web-based population risk-monitoring enterprise dashboards, the effectiveness of the active case finding algorithm has been validated by clinicians and caregivers in Maine.

**Conclusions:**

The active case finding model and associated real-time Web-based app were designed to track the evolving nature of total population risk, in a longitudinal manner, for ED visits across all payers, all diseases, and all age groups. Therefore, providers can implement targeted care management strategies to the patient subgroups with similar patterns of clinical histories, driving the delivery of more efficient and effective health care interventions. To the best of our knowledge, this prospectively validated EMR-based, Web-based tool is the first one to allow real-time total population risk assessment for statewide ED visits.

## Introduction

The use of emergency department (ED) services in the United States is growing at an alarming rate [1-3]. Between 2001 and 2008, the annual number of ED visits in the United States grew at roughly twice the rate of population growth [4]. Recent experience from Oregon’s Health Insurance Experiment suggests that increasing patient access to Medicaid without an accompanying strategy to manage the overall insured population may result in a substantial surge in ED utilization [5], including visits for conditions that may be most readily treatable in primary care settings. Presuming a large proportion of ED visits are preventable*,* attention has turned toward strategies to treat patients in less expensive outpatient care settings, and payers are beginning to deny payment for non-urgent ED visits [6].

Improving appropriate use of emergency services is an important strategy for improving health outcomes and controlling health care expenditures [7]. With the increased adoption of electronic medical record (EMR) systems and the development of health information exchanges (HIE) in the United States, health care organizations have better and more comprehensive access to patients’ comprehensive medical histories. Greater use of advanced analytic computing methods on EMR datasets has led to the development of several active case finding algorithms to assess patient risk. Early efforts included risk prediction models for hospital readmission [8] and repeated ED visits for patients with distinct patterns [9-11]. Most risk development studies focused on patients within specific payer groups, for example, Medicare, within specific age, and/or within specific disease groups [12,13].

We previously developed predictive analytics of patient risk of a 30-day return to the emergency department [14]. The 30-day ED revisit risk is intended for hospital emergency room and quality management staff to immediately plan for post-discharge care while the patient is in the emergency room, or shortly thereafter. This particular risk is triggered by the event of an emergency room visit, and therefore is a very small subset of the whole population, that is, only those patients with at least one emergency room visit are covered. Second, emergency room revisit rates are a quality measure used to assess hospital performance.

In this paper, we describe our findings for the ED visit risk modeling for the statewide population at large, whether or not they have had a previous emergency room visit. This is the first effort to model total population ED risk across all payers, all diseases, and all age groups. Our efforts include the statistical learnings from all Maine HIE patient data contained in the statewide HIE of longitudinal patterns to identify risk factors that strongly influence the probability of a future 6-month ED visit.

**Figure 1:**
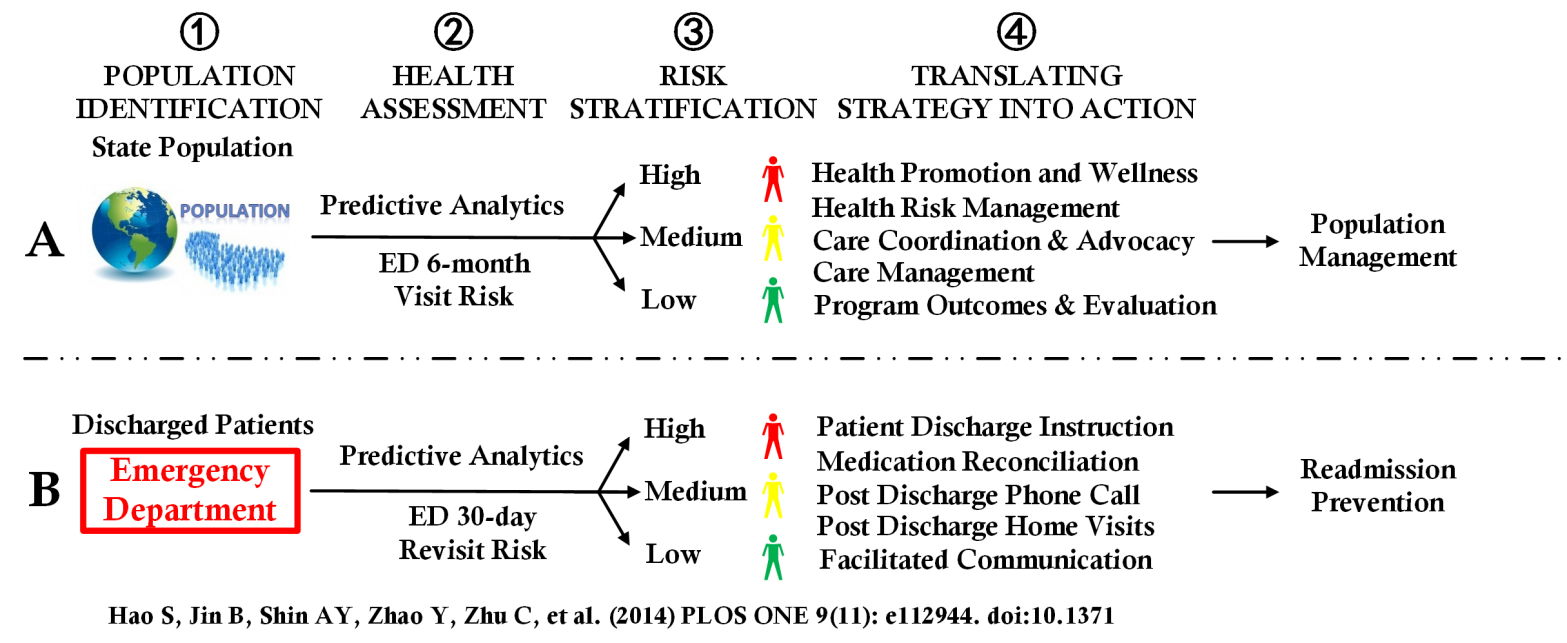
Integrating predictive analytics into workflows of proactive population health management and hospital quality improvement; emergency department (ED) visit risk determination and proactive interventions guided by ED visit risk or ED readmission risk measures.

Although the two metrics (ie, risks of the 30-day ED revisit [14] and the future 6-month ED visit), have similarities in regard to ED visit risk, these are two distinct risks for two distinct purposes (Figure 1). We studied both to understand differences and similarities between them. The population 6-month ED visit risk is intended for the care team responsible for population health management in accountable care organizations (ACOs) and providers with capitated risk contracts.

We hypothesized that real-time assessment of population ED risk to track and trend risk over time can allow health managers to continuously assess and intervene on both high-risk and rising-risk patients. To empower the visualization and exploration of the total population risks of over one million patients in the state of Maine, Web-based apps were designed, aiming to connect in real-time, aggregate, and centrally integrate data, and to compute future 6-month ED risks for population health management.

## Methods

### Ethics Statements

This work was done under a business/product development arrangement between HealthInfoNet (HIN) and HBI Solutions, Inc., and the data use was governed by a business agreement between HIN and HBI. No patient health information was released for the purpose of research and no patient consent was required. We completed the system development that was the foundation for our agreement and then reported on the findings resulting from applying this model to the real-time Web-based services that HIN is now deploying in the field. Because this study analyzed de-identified data to develop the ED risk model, the Stanford University Institutional Board considered it exempt (October 16, 2014).

### Population

The objective was to study total population risk for ED visits across all payers, all diseases, and all age groups. Patients visiting any HIN-connected facility from January 1, 2012 through December 31, 2013 were eligible for study. Patients who died, as identified through an encounter disposition code, were excluded during the study time frame of 2012 and 2013. ED visits transferred from another ED were excluded as these were treated as one ED visit, and not multiple.

### Data Acquisition and Marshalling

We constructed an enterprise data warehouse consisting of all of Maine’s HIE aggregated patient histories. Incorporated data elements from EMR encounters included patient demographic information, laboratory tests and results, radiographic procedures, medication prescriptions, diagnosis, and procedures, which were coded according to the International Classification of Diseases, 9th Revision, Clinical Modification (ICD-9-CM). Census data from the US Department of Commerce Census Bureau were integrated into our data warehouse. Therefore, in addition to the HIN features, we categorized patients by socioeconomic status using residence zip codes as an approximation to the average household mean and median family income and average degree of educational attainment.

Maine HIE patient clinical histories were organized as hospital episode level relational database tables. We processed the database at patient level based on medical record number for population analysis within 36 facilities in Maine. A pivot table was developed from our enterprise data warehouse, which aggregated and integrated normalized clinical features (n=33,403) of different data categories, for example, primary diagnosis/procedure, secondary diagnosis/procedure, laboratory test result, radiology result, and outpatient prescription, from different relational EMR databases. For qualitative and categorical parameters, dummy variables were created serving as numerical representations of the categories of nominal or ordinal variables. To efficiently eliminate the least representative features, we exploited the data variance as the simplest criterion [15], which essentially projected the data points along the dimensions of maximum variances. One potential limitation was that variance alone does not account for parameters that had a small dynamic range. However, as an initial filter, this method effectively eliminated “low information content” features to deliver a manageable feature set, allowing the subsequent machine learning step to identify discriminant features. As a result, a set of patient clinical historical features in the prior 12 months was compiled (Multimedia Appendix 1). One of the key features was whether the patient had a chronic medical condition. This feature was defined using the Agency for Healthcare Research and Quality Chronic Condition Indicator [16], which provides an effective way to categorize ICD-9-CM diagnosis codes into one of two categories: chronic and non-chronic.

### Outcome Time Frame for Risk Analysis

A “time-to-event” curve of ED visits (Multimedia Appendix 2) was developed to determine whether 6-month ED visit assessment was clinically reasonable. More than 80% of patients with more than one ED visit history would seek ED services within the future 6-month time frame. Therefore, future 6-months was a clinically appropriate cutoff. This was in line with clinical and field caregiver judgments.

### Data Mining Overview: Retrospective and Prospective Analyses

**Figure 2:**
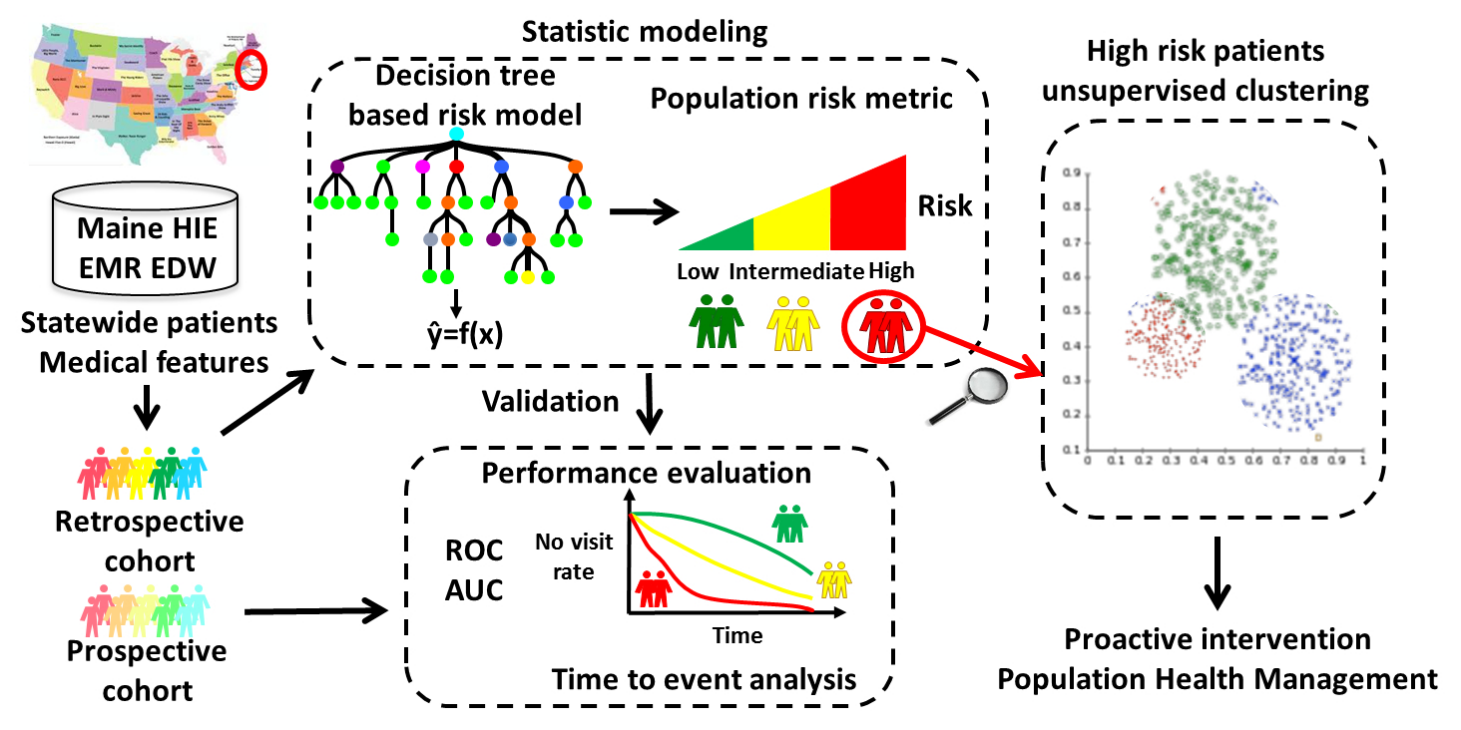
Study design to develop the active case finding algorithm to predict future 6-month emergency department visit risks.

The basic principle of our model was using information of 1 patient in the prior 1 year to predict if this patient would have any ED visit in the next 6 months. The statistical learning to forecast future 6-month ED visit risk consisted of two phases: retrospective modeling and prospective validation (Figure 2). A retrospective cohort of 829,641 patients (Multimedia Appendix 3) who had historical encounter records from January 1 to December 31, 2012, was assembled for the development of the ED risk model to predict if those patients would have ED visits in the next 6 months (January 1 to June 30, 2013). This model was later validated with a prospective cohort of 875,979 patients (Multimedia Appendix 3) who had historical encounter records from July 1, 2012, to June 30, 2013 to predict if these patients would have ED visits in the next 6 months (July 1 to December 31, 2013). Both cohorts of patients had comprehensive clinical histories allowing the determination of future 6-month ED visit risk. Patients in the retrospective and prospective cohorts were similar in age, gender, income, and education, as well as incidence of future 6-month ED visits (retrospective: 11.48%, 95,241/829,641; prospective: 11.37%, 99,558/875,979) (see Multimedia Appendix 4).

### Retrospective Analysis Summary

The goal of this study was to develop an active case finding algorithm with a statewide future 6-month ED visit risk measure. The measure comprised a single summary score, derived from the results of a “forest” of the most discriminative decision trees upon 1 year of the encounter history. The measure calculated each subject’s probability of a future 6-month ED visit. The retrospective modeling phase consisted of three steps: (1) training, (2) calibrating, and (3) blind testing. We applied a selective cohort division process while trying to result in a random cohort. The samples in the retrospective cohort were divided into six subgroups based on histories of chronic diseases, historical ED visits, and current primary diagnoses (Multimedia Appendix 3). Then, in each subgroup, the case (future 6-month ED visit counts > 0) and control (future 6-month ED visit count=0) samples were randomly partitioned into three cohorts (Cohort I: training cohort, Cohort II: calibrating cohort, Cohort III: blind testing cohort), with the consideration that the past 12-month ED histories of encounters and principle clinical features (chronic diseases and current primary diagnoses) achieved a balance between the cohorts. Therefore, it was hard to achieve a complete balance such that total samples in training, calibrating, and blind testing cohorts had the exact same number. Within each subgroup sharing balanced numbers of chronic histories, ED visits, and current primary diagnoses, the patient numbers in training, calibrating, and blind testing cohorts were close.

### Decision Tree-Based Modeling

A “survival forest” of forecasting decision trees was developed using the prior year clinical history and was ranked according to the corresponding posterior probability. To introduce the prior knowledge, we grouped the clinical features into two groups: empirical features found by exploratory data analysis and the learned features discovered during the model training. Our exploratory analysis (Multimedia Appendix 5) of the retrospective cohort showed that the percentage of patients with future 6-month ED visits increased as a function of either historic ED visit counts or the presence of chronic disease diagnoses; therefore, these two features were strongly associated with patients’ risk for future 6-month ED visits. Using empirical features of whether patients had historic ED visit or a chronic disease diagnosis in the prior year, we built a decision tree. This deterministic tree partitioned the Cohort I samples into four subgroups. Within each subgroup, learned features were discovered through the feature selection process to develop the correspondent learning model for the targeted subgroup. Survival tree analysis was applied to learning model process to predict ED visit day after predicted time. Technical details of the model training process [14,17,18] are presented in Multimedia Appendix 6.

### Risk Scoring Metric Development

Cohort II was used to calibrate the predictive scoring threshold to create a risk measure for each individual sample. Applying the model developed with Cohort I to each sample in Cohort II, the derived predictive scores were ranked. After this, we applied a mathematic function mapping predictive values (PPVs).

Our active case finding algorithm was set to segregate the population into subgroups with different levels of future 6-month ED risks. The risk measure was defined as an index between 0 and 100 so that the people with measures larger than or equal to a risk index *L* had a probability of *L*% to have an ED visit in the next 6 months. Here, the mapped PPV was defined as the individual’s risk measure for the future 6-month ED visit.

We obtained two thresholds, *T*_*h*_ *,T*_*m*_ *,* from this mapping. The intent of the model was to stratify the patients from low to high risk allowing the clinicians to target different risk levels for personalized intervention. Field care providers can target different risk groups with different threshold settings as a continuous variable for active case finding. Two thresholds of 0.3 and 0.7 were chosen and applied to the ranked outputs of the model to divide the population into low (score<30%), medium (score≥30% and score<70%), and high (score≥70%) risk groups [7].

### Identification of the Discriminant Features

In our implementation, the objective was to select the least number of representative features predictive of future 6-month ED risk and to achieve optimal case finding sensitivity while maintaining the targeted PPV (>70%) based on selected features (Multimedia Appendix 7, left panel). The active case finding algorithm identified 101 variables (Multimedia Appendix 7, right panel) predictive of future 6-month ED risk, which fell into the following general categories: age groups (n=4), history of different encounter types (n=8), history of primary (n=17) and secondary (n=8) diagnosis, specific chronic diseases (n=8), laboratory test results (n=28), history of radiographic tests (n=3), and history of outpatient prescription medications (n=25). The predictive power of the selected features was examined by shrunken difference [19] (Retrospective: Multimedia Appendix 7, right panel; Prospective: Multimedia Appendix 7), which was the scaled distance between the mean values of each feature variable in a specific risk class (low, medium, or high) and across all cohort samples. Shrunken differences among the low-, medium-, and high-risk outcomes differed more than the case (with future ED) and control (without future ED) outcomes, demonstrating the effectiveness of these features in the risk stratification.

### Blind Testing

Cohort III was an independent naive sample set, which was compiled to blind test the active case finding method’s performance. The aim of this step was to critically assess the utility of the risk measure before statewide prospective validation in Maine. Again the model developed with Cohort I was applied to every sample in Cohort III to derive and rank the predictive scores and calculated the receiver operating characteristic (ROC) area under the curve (AUC) score.

### Prospective Validation

The clinical application of the future 6-month risk measure was deployed for prospective validation on the HIE data in Maine. The cohort of 875,979 patients from July 1, 2012 to June 30, 2013 was prospectively profiled to calculate future 6-month ED visit risk measures using the clinical applications deployed at HIN. The ROC [20] and time-to-event analyses were performed to gauge the model performance (Multimedia Appendix 8) and effectiveness of the risk stratification.

### Unsupervised Clustering: Subgroup Analysis of High-Risk Patients

We used principle component analysis [21] to reduce high dimensional EMR features and identify clinically relevant groups of patients of high risk for 6-month ED visit with similar patterns of demographics, primary diagnosis and procedure, and chronic disease conditions. The features for high-risk patients were projected to a lower dimensional subspace with largest variances. The *K*-means algorithm was applied to find potential patient patterns for future 6-month ED visit [22]. We used *K*=6 to generate the final six clusters. The technical details are described in Multimedia Appendix 9. Clustering patterns between retrospective and prospective cohorts were compared to further validate our high-risk case finding algorithm. As part of the health care management platform, our predictive model was integrated onto a Web-based dashboard to provide a real-time visualization of the population profile with ED 6-month visits.

### Population Explorer Service: Statewide Real-Time Surveillance of Population ED Risks

The active case finding model and associated real-time Web-based app were designed to track the evolving nature of total population risk, in a longitudinal manner, for ED visits across all payers, all diseases, and all age groups. Patient historical datasets are linked and stored in a patient-level database in our system. ED predictive algorithm is applied to the individual’s ED discriminating feature data to risk-stratify the patients with our prospectively validated model. Individual data are then aggregated for population exploration of ED risks, which are stored in the population-level database. Our dashboard allows the visualization of the population ED risks at high geographical resolution for a defined population, for example, the population of Maine.

## Results

### Data Mining Overview: Retrospective and Prospective Analyses

**Figure 3:**
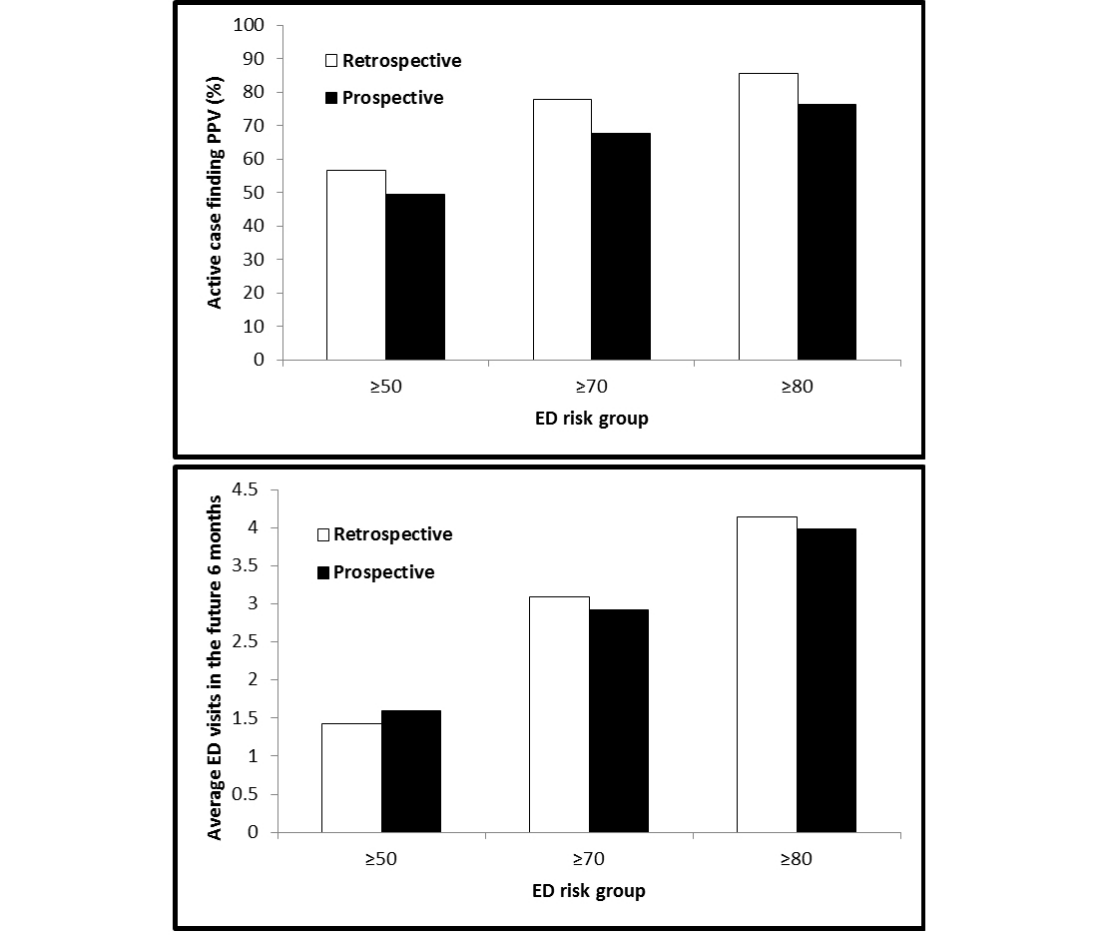
Active case finding algorithm effectively identified different risk group patients for future 6-month emergency (ED) utilization (upper panel shows X axis: different ED risk groups; Y axis: active case finding positive predictive value (PPV); and lower panel summarizes average ED uses at different ED risks in the future 6 months in both retrospective and prospective analyses).

The active case finding algorithm produced a risk score (from 0 to 100) for each patient at the time of risk assessment of the future ED visit. In general, our algorithm achieved high performance that ROC AUCs of the risk score for a determination of risk of patient future 6-month ED utilization were 0.739 and 0.696 in retrospective blind testing and prospective validating respectively (Multimedia Appendix 8). Specifically, at a risk score threshold of 50, the active case finding algorithm identified 56.55% (9459/16,727) of retrospective and 49.35% (10,810/21,904) of prospective patients who had an ED visit in the next 6 months; 43.45% (7268/16,727) of retrospective or 50.65% (11,094/21,904) of prospective patients were identified incorrectly (who did not have an ED visit) (Figure 3). At risk score threshold levels of 70 and 80, the rate of incorrectly “flagged” patients dropped to 22.20% (839/3780) and 14.48% (286/1975) in retrospective, and 32.31% (1764/5460) and 23.69% (626/2642) in prospective analysis respectively, but the algorithm found a lower percentage of patients. The ROC analyses showed that there was a 0.739 (retrospective) or 0.732 (prospective) probability that a randomly selected patient with a future 6-month ED visit would receive a higher-risk score than a randomly selected patient who did not have a future 6-month ED visit.

### Prospective Validation

**Figure 4:**
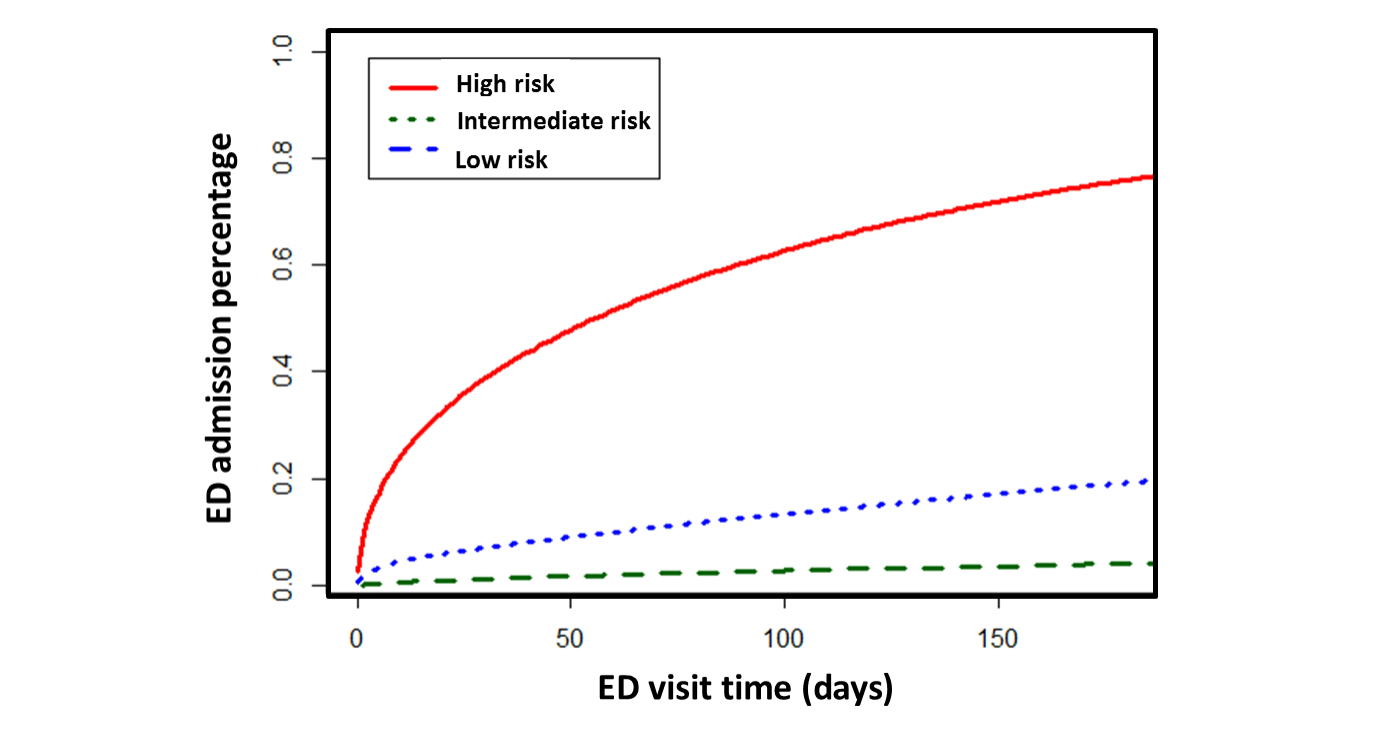
Active case finding algorithm effectively risk-stratified the prospective patient cohort for future 6-month emergency department (ED) visit (graphic representation of low, medium, and high risk patients’ time to next impending ED visit).

In developing the algorithm, we aimed to help potential care providers assess the “opportunity case” (high-cost, high degree of utilization of services, multiple chronic conditions) for various risk score thresholds and for different assumptions about the impact of the intervention. The active case finding algorithm was capable of stratifying patients across a wide range of risks (Figure 3, upper panel) and demonstrated that patients in higher-risk categories visited the ED earlier (prospective time-to-event analysis: *P*<.001) both on retrospective (Multimedia Appendix 10) and prospective (Figure 4) cohorts, and more frequently (Figure 3, lower panel) over the future 6-month period.

### Unsupervised Clustering: Subgroup Analysis of High-Risk Patients

Our principle component analysis retrospectively identified (Multimedia Appendix 11, left panel) and prospectively confirmed (Multimedia Appendix 11, right panel) a pattern of six distinct subgroups among the high-risk patients with risk scores greater than 70. These six clinically relevant clusters (retrospective: Multimedia Appendix 12, prospective: Multimedia Appendix 12) grouped around multiple “anchoring” demographic and chronic disease conditions. The chronic conditions co-occurred in many instances and included endocrine, nutritional, and metabolic diseases as well as immune disorders (ranging from 23.83%, 245/1028 to 74.21%, 590/795), diseases of the circulatory system (ranging from 13.7%, 99/722 to 68.4%, 544/795), diseases of the nervous system and sense organs (ranging from 26.0%, 188/722 to 66.5%, 529/795), diseases of the respiratory system (ranging from 23.44%, 241/1028 to 50.6%, 402/795), and diseases of the digestive system (ranging from 17.41%, 179/1028 to 55.0%, 437/795). These conditions were prevalent in all clusters, indicating that endocrine, immune, cardiac, nervous, respiratory, and digestive system dysfunctions co-occur. The largest cluster (#1) was characterized by predominantly adult female patients (between the ages of 19 and 49) characterized by chronic conditions including endocrine, nutritional, metabolic, and immune disorders, diseases of the sense organs, nervous, digestive, and respiratory systems. Cluster #6 was revealed as a high resource-consuming subgroup with the largest number of distinct chronic disease diagnoses accompanied by the largest number of laboratory and radiographic tests. In contrast, Cluster #5 contained a relatively younger population (age 19 to 34) with diminished incidence of chronic disease and minimal resource consumption. Clusters #2, 3, and 4 shared similar age, gender, and chronic disease distributions; however, these clusters displayed different usage profiles for tests and medication prescriptions. Moreover, in Clusters #2 and #3, approximately 20% of patients did not have any chronic disease diagnosis in the prior 12 months. Among Clusters #1 through #4, there are no direct correlations between the number of distinct chronic disease diagnoses and the usage of tests and prescriptions.

### Population Explorer Service: Statewide Real-Time Surveillance of Population Emergency Department Risks

**Figure 5:**
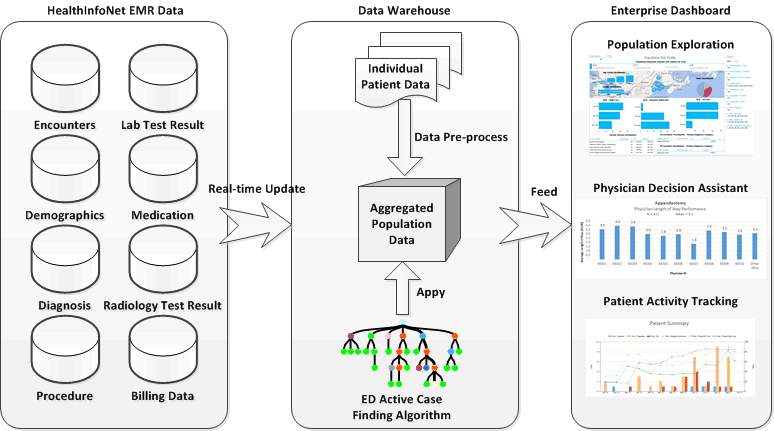
Schematic demonstration of data flow and communications of a population emergency department (ED) risk exploration system that allows real-time assessment of population ED risk.

**Figure 6:**
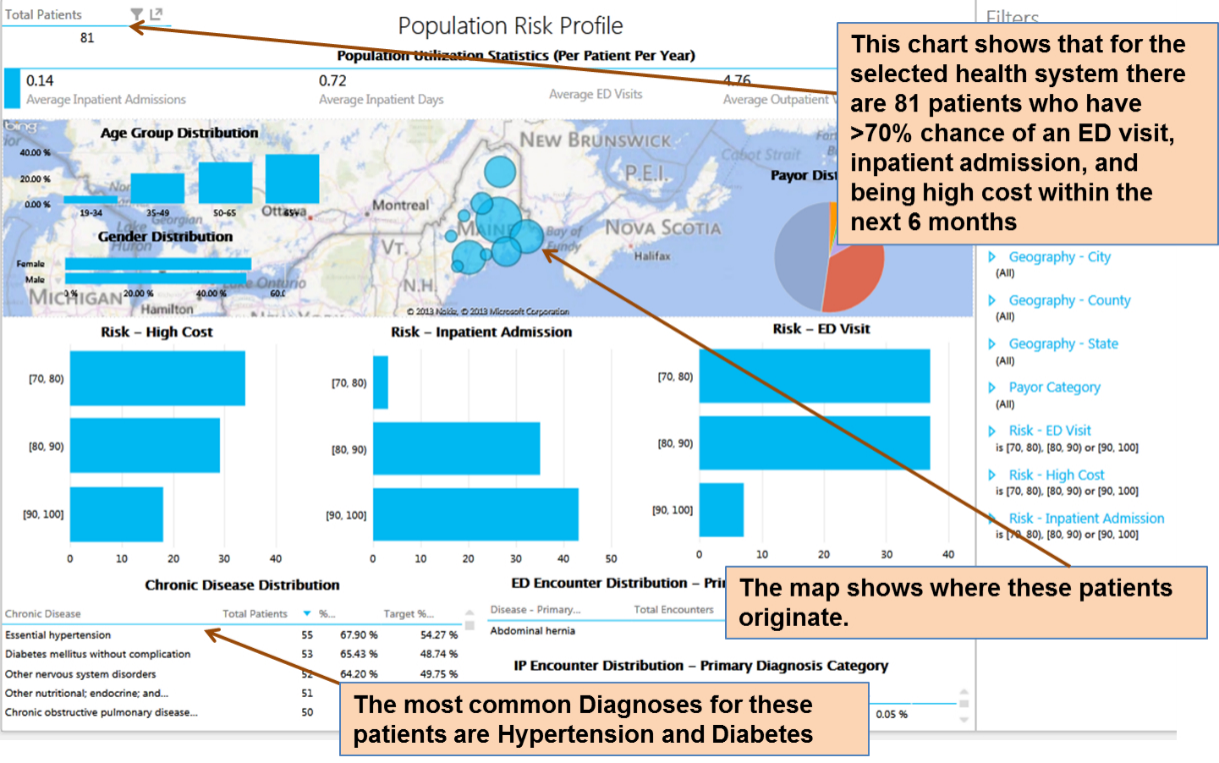
Total population emergency department (ED) risk monitoring dashboard.

Our ED predictive analytics were integrated into the Maine State HIE system (Figures 5 and 6) to allow real-time surveillance of population ED risks. Triggered by real-time iterative data feeds, each patient’s ED risk measure can be updated periodically upon new data feed. This allows for trending risk scores over time, whereby targeting patients with major increases in risk may be as useful as targeting the patients at the highest risk. This Web-based population risk surveillance dashboard (Figure 6) empowers the ACO field staff and population health managers to visualize the ED risks derived from each resident’s historical medical records in Maine. With our prospectively validated ED risk case finding algorithm, our coherent view of population ED risks can thus be feasible to resolve the barrier of the fragmented nature of population health information to improve public health practice and reduce ED utilization.

## Discussion

### Principal Findings

We hypothesized that an individual patient’s future 6-month ED risk can be forecast from the statistical learning of a population’s comprehensive longitudinal clinical histories. Our use of the population-based HIE facilitated the development and prospective testing of the case finding algorithm presented here, which is population-based and not event-triggered (ED visit) analytics. After calculating the total population risk scores for future ED visit risk scores, this information is then made available to clinicians and caregivers at the point of care to support both individual patient and population-based decision making. Using adjustable risk settings allows multiple patient cohorts of different impending ED risks to be constructed. Moreover, high-risk patients with similar longitudinal clinical patterns can be subgrouped for targeted intervention in real time. Accurate identification of patient populations at high risk for ED visits is an integral component to address specific gaps in health care coverage, institute primary care-based interventions, and avoid preventable ED visits. Such active case finding may help providers deliver more efficient and effective health care interventions.

### Strengths and Limitations

Designed to be used in real time by population panel managers to forecast a future ED visit, our EMR-based active case finding method was prospectively validated with a reasonable level of sensitivity and specificity. To the best of our knowledge, our EMR-based population ED risk study is the first with such scale for ED trending across all payers, all diseases, and all age groups. Our study’s obvious strength is the use of an entire US state in regard to predictive analytics. Its weakness is the study cohort’s potential patient characteristics unique to the state of Maine, which may limit our model’s general applicability to the other state populations.

Data limitations, for example, missing data, inaccurate diagnostic/procedure coding, and the unreliable tracking method to identify patients who die, may result in false negative and false positive case calls. Additionally, new patients who lack encounter histories tended to be categorized as low risk for future ED visits, a function that likely underestimates the ED risk for these subjects. We speculate that using additional currently non-reported features, including self-rated health conditions, lifestyle-related factors, and socioeconomic status may enhance the analytical approach to ED visit risk assessment.

Beyond identifying at-risk populations for potentially preventive services, gaining a deeper understanding of both the unique and common attributes of various subgroups may further facilitate overall management and the prevention of unwanted ED utilization. Moreover, to be clinically useful, a case finding model should be iterative and facilitate exploration of the potential benefit (PPV) or burden (false positive rate) (business case) of managing subpopulations of high-risk patients. Accordingly, we sought to determine whether unique patterns of resource utilization or clusters of patient subpopulations existed among the considerable heterogeneity of the high-risk patient population when considered together. We demonstrated that among the high-risk group patients, their associated demographics, chronic conditions, and varying patterns of resource consumption do not occur in isolation. Cluster analysis revealed six clinically relevant subgroups among the high-risk patient population that were confirmed as durable upon prospective testing. These subgroups have unique patterns of demographics, disease severities, comorbidities, and resource consumption, suggesting new opportunities to provide stratified care management among these groups. For example, Cluster #6 had senior patients with co-occurring histories of the most diverse chronic conditions and linked to the highest utilization of clinical tests and prescriptions. In addition, this group of patients is at considerable risk to experience poor health outcomes, including, but not limited to, lower quality of life, diminished functional status, as well as excess morbidity and mortality. This distinctive cluster could be targeted with new, enhanced care management strategies. We noted a decreased prevalence of the co-occurring chronic conditions in four other cluster groups of relatively younger adults with much less resource consumption. Within these four clusters, females aged 19-49 years without any chronic disease may benefit from targeted care to keep them out of the emergency room, although more analysis is needed to understand the risk drivers within this group. Currently, many existing care management strategies are directed toward single conditions and are event-triggered, for example, ED visit or hospital discharge. The current active case finding model provides novel opportunities to experiment with new strategies of coordinated care targeting a combination of conditions across different age and demographic groups that we speculate may lead to greater case management efficacy.

While the clusters identified in the study represent clinically similar populations that could guide specific care management strategies, we understand that the missing information (eg, mental health and substance abuse diagnostic information) may mask important characteristics of these clusters. Past studies have shown that mental health diagnoses are frequent within the ED patient population [23]. With data quality improving over time, we see a future opportunity for overall improvement in the predictive model and subsequent patient clustering.

With our ED risk model, tactics for modifying care management programs can be driven and measured against the analytical risk assessment derived from the HIE records. HIEs are a valuable data resource, providing longitudinal and comprehensive patient data. HIEs, such as HIN, that have completed the necessary rigorous mapping of multiple providers’ data to standard nomenclature including LOINC [24], RXNorm [25], and SNOMED [26] offer an unparalleled data repository that can be leveraged to realize value through the application of advanced analytic techniques. However, while HIE data represents an ideal source of community-wide/regional patient data, operational HIEs are not present in all states. The predictive model and patient clustering method can be applied to any clinical dataset including the clinical EHRs directly as well as private HIEs within hospital networks.

### Conclusions

Our study is the first study of total population risk for ED visits across all payers, all diseases, and all age groups. Applying analytical tools on EMR and HIE data, including the active case finding model, the high-risk patient clustering method, and the Web-based real-time ED risk profiling analysis and exploration, will help health care providers effectively leverage their EMR to better understand ED service delivery while providing opportunities for improved health care delivery for the patients. A great strength of this work is the use of data from an entire state HIE, including data from across the entire spectrum of the health care system. This is not just hospital or emergency department data because it includes outpatient clinics and physician practices. In that regard, our work should serve as a model of what other states can do with HIE data to really impact patient care and population health.

## Multimedia Appendix 1

Electronic medical record (EMR) features used to develop active case finding model.

## Multimedia Appendix 2

Emergency department (ED) admission “time-to-event curve” showed pattern of rapid accrual with stable and consistent ED visit rate thereafter. Population ED visit curves, of patients with more than one or any ED visit, stabilized within 6 months from evaluation time, indicating a 6-month cutoff is clinically reasonable. Assessment date: January 1, 2013.

## Multimedia Appendix 3

Study cohort construction, and inclusion/ exclusion criteria; retrospective/ prospective cohort construction.

## Multimedia Appendix 4

Patient characteristics.

## Multimedia Appendix 5

Exploratory data analysis: patient counts of total set and those having emergency department (ED) revisit in future 6 months, as function of number of chronic diagnoses (left panel) and ED visits in past 12 months (right panel), and percentages of patients with ED revisits was also plotted.

## Multimedia Appendix 6

Technical details of decision tree based modeling.

## Multimedia Appendix 7

Feature selection and characterization of discriminant features in retrospective/prospective dataset.

## Multimedia Appendix 8

The model performance was gauged by ROC analysis for retrospective blind testing and perspective validating respectively.

## Multimedia Appendix 9

Unsupervised clustering of high risk population using principal component analysis.

## Multimedia Appendix 10

Active case finding algorithm effectively risk-stratified retrospective patient cohort for future 6-month emergency department (ED) visit: graphic representation of low-, medium-, and high-risk patients’ time to the next impending ED visit.

## Multimedia Appendix 11

Unsupervised clustering of the high-risk patients identified consistent distinct subgroups in both retrospective (left panel) and prospective (right panel) cohorts.

## Multimedia Appendix 12

Clustering of emergency department 6-month high-risk patients in the retrospective/prospective cohort according to demographics and prior year clinical histories.

## Abbreviations

ACO: accountable care organization
AUC: area under the curve
ED: emergency department
EMR: electronic medical record
HIE: health information exchange
HIN: HealthInfoNet
ICD-9-CM: International Classification of Diseases, 9th Revision, Clinical Modification
PPV: positive predictive value
ROC: receiver operating characteristic
