# Supplementary material for: Real-Time Web-Based Assessment of Total Population Risk of Future Emergency Department Utilization: Statewide Prospective Active Case Finding Study
Source: Interact J Med Res. 2015 Jan 13;4(1):e2. doi: 10.2196/ijmr.4022 (PMC4319080; doi:10.2196/ijmr.4022)
Supplement: Supplementary file 1 [file ijmr_v4i1e2_app1.pdf]

Multimedia Appendix 1. EMR features used to develop the ACF model.

Feature group	Number of Feature	Feature description (in the past 12 months)
Encounter history	170	Visit counts of different encounter types (E/O/I/P/R) [a]
		The accumulated length of hospitalized stay
		Counts of historical chronic disease diagnoses
		Counts of total and no redundant total radiographic and laboratory tests, and medication
Demographics	10	Female, male
		Age group is defined by age at admission
		(0, 1-5yr, 6-12yr, 13-18yr, 19-34yr, 35-49yr, 50-65yr, 65+yr)[b]
Diagnosis	1582	Counts for each primary diagnosis and secondary diagnosis
Procedure	45	Counts for each primary procedure and secondary procedure
Comorbidity	27	Counts for comorbidity diseases
Radiographic test	159	Counts for each radiographic test
Laboratory test	1023	Counts for each laboratory test results
Outpatient prescription	1527	Counts for each outpatient prescription

[a] Encounter type description: E – Emergency, O – Outpatient, I – Inpatient, P – Pre admission, R – Recurring admission

[b] yr – year
