# Supplementary figures and images for: Real-Time Web-Based Assessment of Total Population Risk of Future Emergency Department Utilization: Statewide Prospective Active Case Finding Study
Source: Interact J Med Res. 2015 Jan 13;4(1):e2. doi: 10.2196/ijmr.4022 (PMC4319080; doi:10.2196/ijmr.4022)

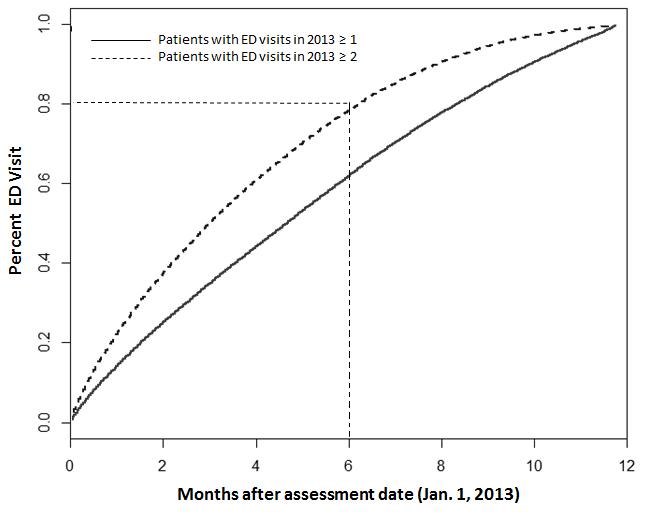

Supplement: Supplementary file 2 [file ijmr_v4i1e2_app2.png]

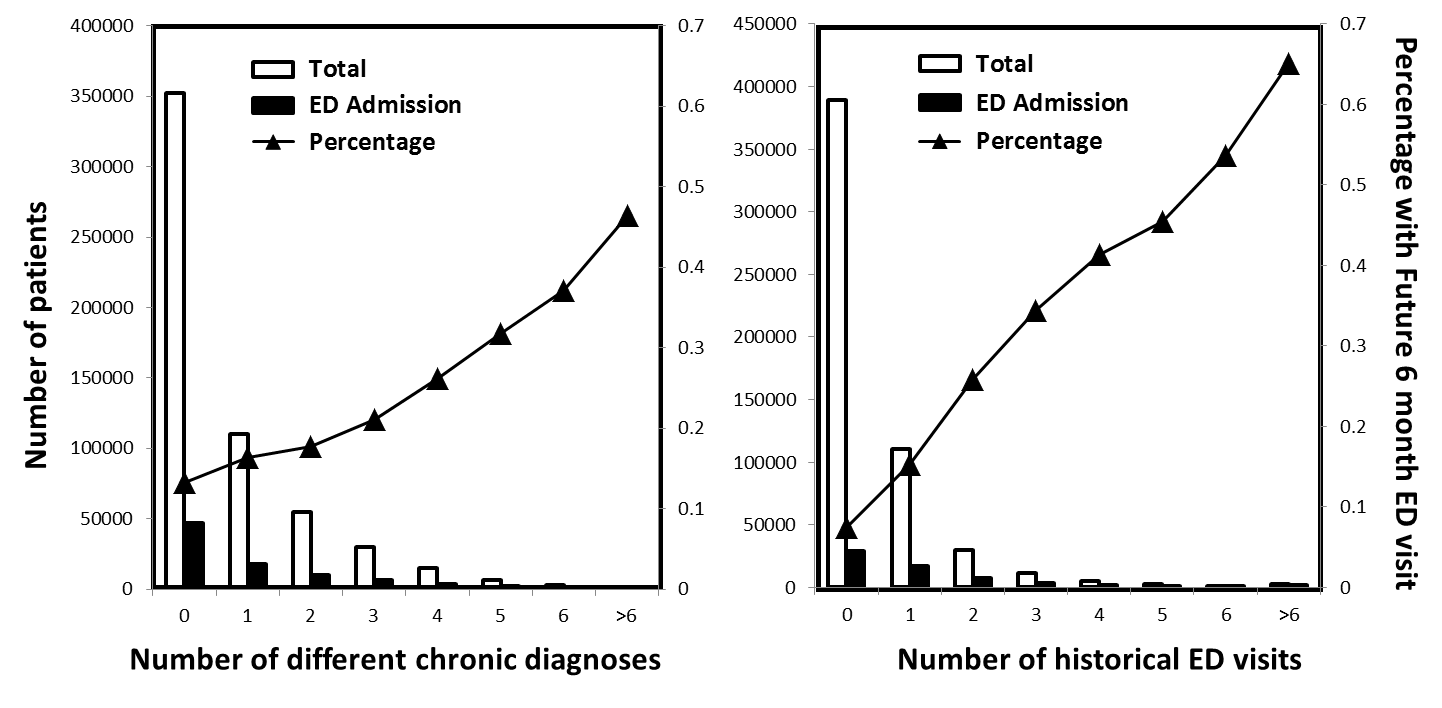

Supplement: Supplementary file 5 [file ijmr_v4i1e2_app5.png]

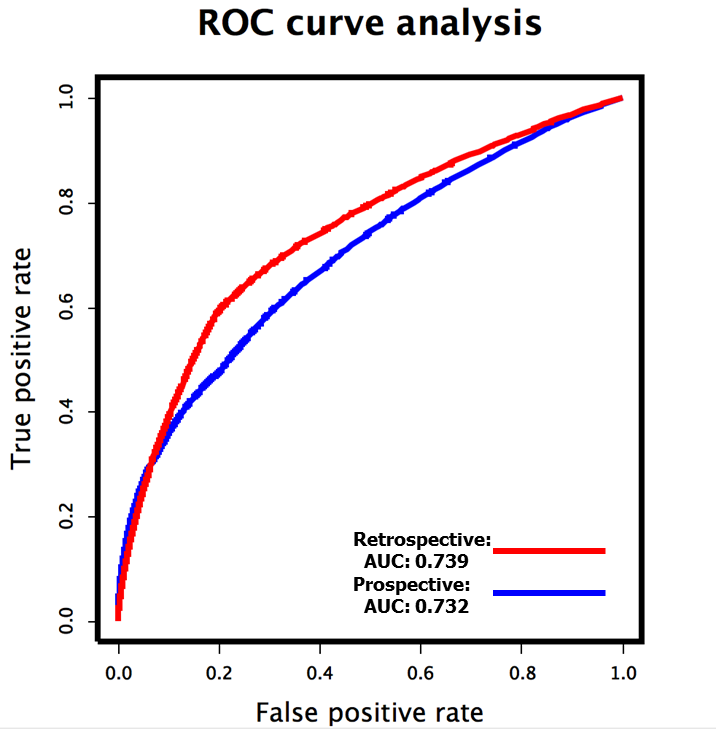

Supplement: Supplementary file 8 [file ijmr_v4i1e2_app8.png]

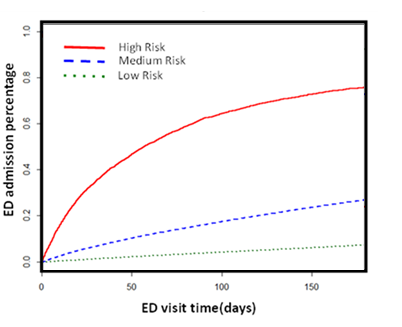

Supplement: Supplementary file 10 [file ijmr_v4i1e2_app10.png]

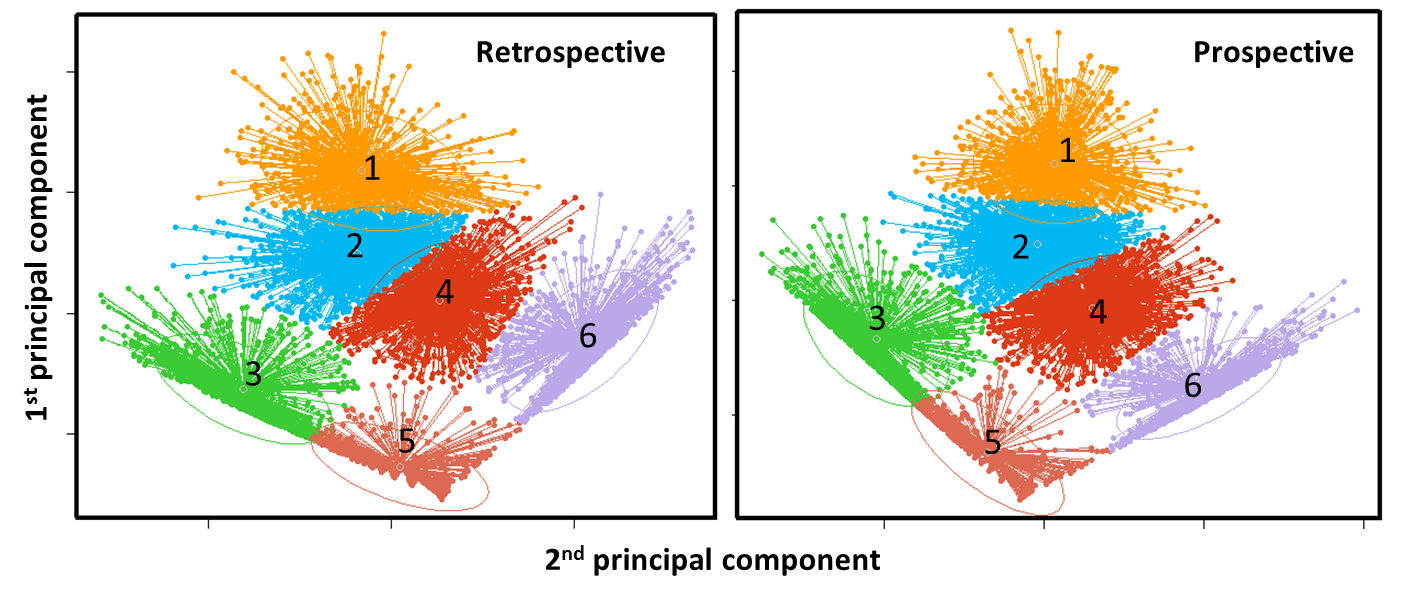

Supplement: Supplementary file 11 [file ijmr_v4i1e2_app11.png]
