# Supplementary material for: Real-Time Web-Based Assessment of Total Population Risk of Future Emergency Department Utilization: Statewide Prospective Active Case Finding Study
Source: Interact J Med Res. 2015 Jan 13;4(1):e2. doi: 10.2196/ijmr.4022 (PMC4319080; doi:10.2196/ijmr.4022)

Multimedia Appendix 3. Study cohort construction, and inclusion/exclusion criteria: Retrospective cohort construction.

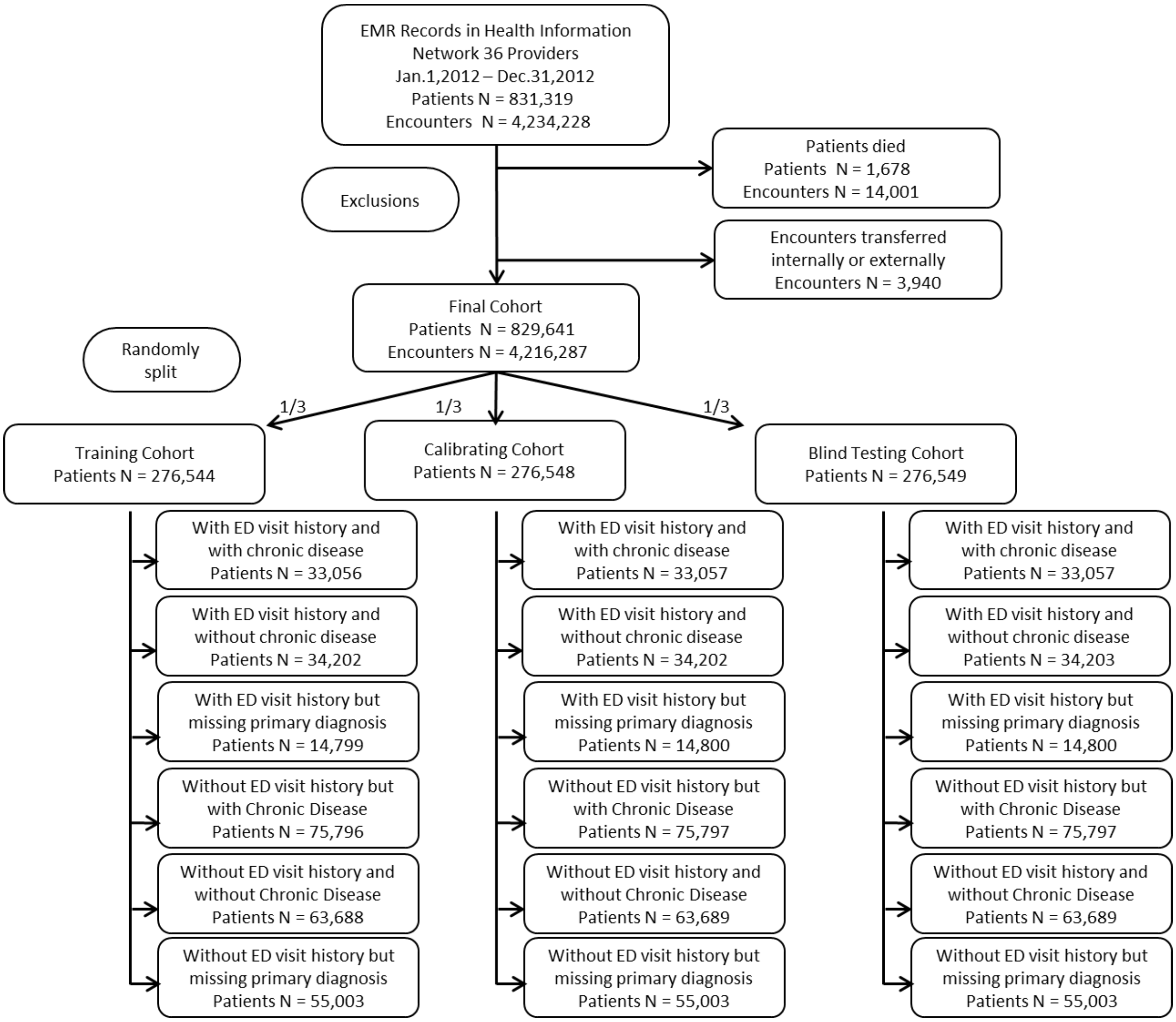

Study cohort construction, and inclusion/exclusion criteria: Prospective cohort construction.

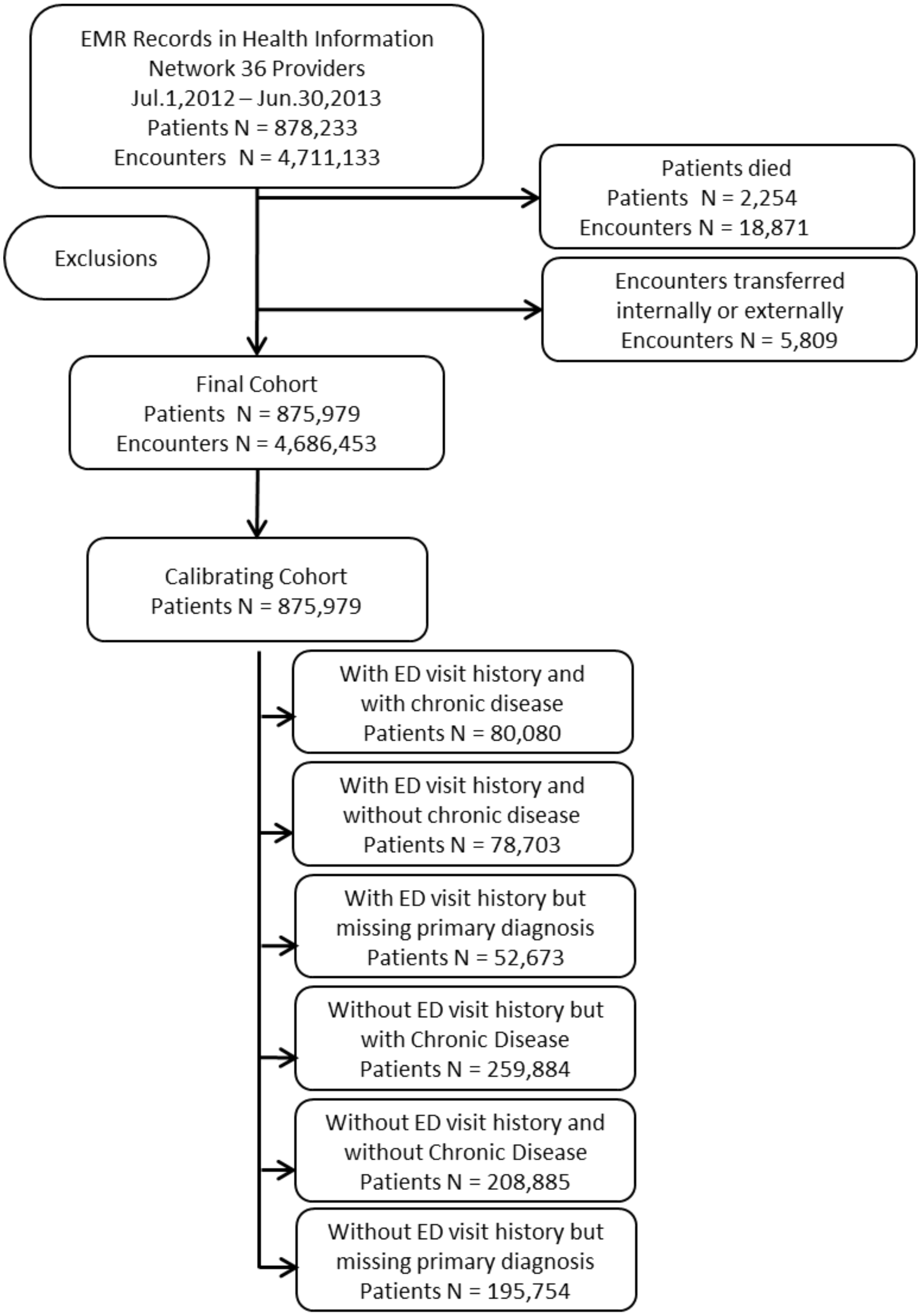

Supplement: Supplementary file 3 [file ijmr_v4i1e2_app3.pdf]
