# Supplementary material for: Real-Time Web-Based Assessment of Total Population Risk of Future Emergency Department Utilization: Statewide Prospective Active Case Finding Study
Source: Interact J Med Res. 2015 Jan 13;4(1):e2. doi: 10.2196/ijmr.4022 (PMC4319080; doi:10.2196/ijmr.4022)
Supplement: Supplementary file 4 [file ijmr_v4i1e2_app4.pdf]

Multimedia Appendix 4. Patient characteristics.

	**Retrospective (Jan.1, 2012 – Dec.31.2012)**				**Prospective (Jul.1.2012 – Jun.30.2013)**			
	**Control**	**Case**	**Test statistics**	***p* value**	**Control**	**Case**	**Test statistics**	***p* value**
	N = 734400	N = 95241			N = 776421	N = 99558		
**Gender**			Chi square test	< .001			Chi square test	< .001
Female	408326(55.6%)	53811(56.5%)			429787(55.4%)	55710(56.0%)		
Male	326074(44.4%)	41430(43.5%)			346634(44.6%)	43848(44.0%)		
**Age**			Ranksum test [a]	< .001			Ranksum test	< .001
median(IQR)	47.9(24.8,63.2)	41(22.5,60.8)			46 (24, 63)	39 (23, 59)		
**Median Family Income Estimate**			Ranksum test	< .001			Ranksum test	< .001
median(IQR)	59510(51140,68140)	57040(48690,65170)			59510(49849, 68138)	57500(47644, 66591)		
**Percent High School Graduate or Higher**			Ranksum test	< .001			Ranksum test	< .001
median(IQR)	90.5(87.4,93)	89.4(86.4,92.4)			90.5(87.3, 93.2)	89.4(86.4, 92.5)		
**Percent Bachelor's Degree or Higher**			Ranksum test	< .001			Ranksum test	< .001
median(IQR)	24.4(18.1,32.4)	22.2(16.7,29)			24.4(18.1, 32.5)	22.2(15.9, 29.1)		

[a] Ranksum test was used when the number of the subcategories violates the assumptions of the Chi square test.
