# Supplementary material for: Real-Time Web-Based Assessment of Total Population Risk of Future Emergency Department Utilization: Statewide Prospective Active Case Finding Study
Source: Interact J Med Res. 2015 Jan 13;4(1):e2. doi: 10.2196/ijmr.4022 (PMC4319080; doi:10.2196/ijmr.4022)
Supplement: Supplementary file 6 [file ijmr_v4i1e2_app6.pdf]

Multimedia Appendix 6. Technical details of decision tree based modeling.

First, a general technique of bootstrap aggregating (bagging) was applied for building multiple decision trees by repeatedly and randomly resampling training cohort with replacement, and voting the trees for a consensus prediction. Second, the survival trees were grown based on the randomly selected predictors via Log-rank survival splitting rule on each survival tree node, which maximizes survival differences across daughter nodes.

Fx,c=i=1Ndi,1-Yi,1diYii=1NYi,1Yi1-Yi,1YiYi-diYi-1di

Here, c is the split value for predictor x; di,j and Yi,j for node h equal the number of patient who visited ED and had no ED visit before time ti in next 6 months period for daughter nodes j=1,2. Hence, Yi,1=#Tl≥ti & xl≤c and Yi,2=#Tl≥ti & xl>c, where Tl is ED visit time in next 6 month for the individual l . The value Fx,c is the measure of node separation, the greater difference between case and control groups and the better the split for the predictor is. Therefore, the optimized predictor x* and split value c* at node h is determined by maximizing the Fx*,c* such that Fx*,c*≥Fx,c for all x and c.

Third, an ensemble cumulative hazard estimate by combining information from the survival trees so that each individual will be assigned one estimate.

Hht=tl,h≤tdl,hYl,h

Where Hht is the cumulative hazard estimate for node h, tl,h is the distinct ED visit times in node h, dl,h and Yl,h represent the number of patients who visited ED and had no ED visit before time tl,h. Hht was computed for terminal node for each predictor xi for individual sample i drop down into in the tree. Therefore,

Ht|xi=Hht, if xi∈h

To compute individual estimate for all trees, ensemble average for all tree cumulative hazard estimate score.

Het|xi=1ntreeb=1ntreeHbt|xi

Here, b denotes the individual tree and ntree is the number of trees in survival forest.
