# Supplementary material for: Real-Time Web-Based Assessment of Total Population Risk of Future Emergency Department Utilization: Statewide Prospective Active Case Finding Study
Source: Interact J Med Res. 2015 Jan 13;4(1):e2. doi: 10.2196/ijmr.4022 (PMC4319080; doi:10.2196/ijmr.4022)
Supplement: Supplementary file 7 [file ijmr_v4i1e2_app7.pdf]

Multimedia Appendix 7.

Feature selection and characterization of the discriminant features in the retrospective dataset.

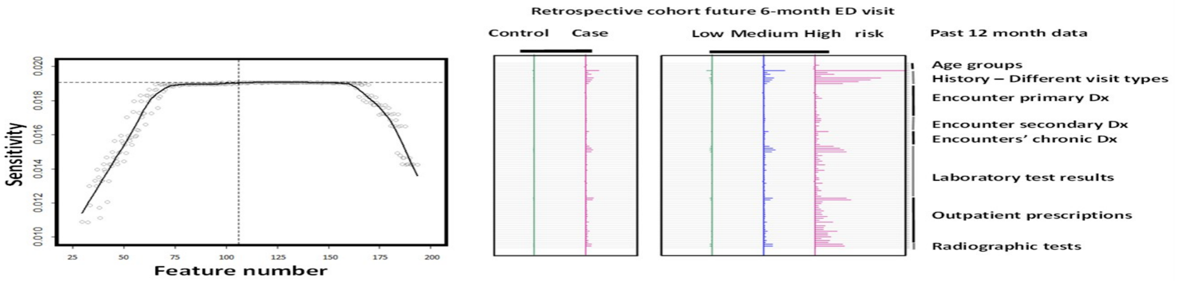

Left panel: We set to select the least number (101 features, dotted vertical line) of representative features predictive of future 6-month ED risk and achieve optimal case finding sensitivity while maintaining the targeted positive predictive value (PPV>70%) based on selected features.

Right panel: Shrunken difference for the selected 101 features to develop the ACF model. Comparing the two cohorts (case/control or the low/medium/high risk), the shrunken differences of these discriminative features were bigger in the low/medium/high risk cohort, demonstrating the effectiveness of these features in retrospectively differentiating these three outcomes.

Characterization of the discriminant features in the prospective data set.

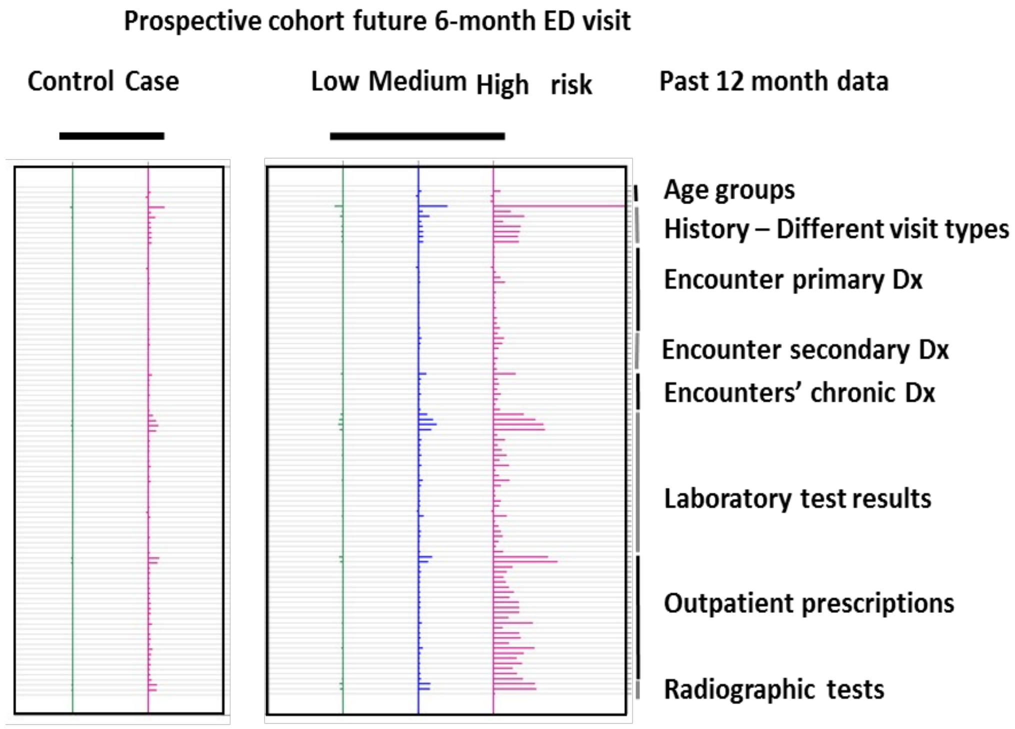

For the features selected during the model development, each feature was ranked with a non-zero value, demonstrating its either positively associated or negatively associated relationship with case. The nearest shrunken centroid (NSC) "shrinks" the centroid to further reduce noise by eliminating weak associations and keeping only those features with strong associations. Shrunken differences for the selected 101 features to develop the active case finding model were graphed. The shrunken differences of these discriminative features in the prospective dataset were bigger in the low/medium/high risk cohort than the case/control cohort, demonstrating the effectiveness of these features in prospectively differentiating these three risk outcomes.
