# Supplementary material for: Real-Time Web-Based Assessment of Total Population Risk of Future Emergency Department Utilization: Statewide Prospective Active Case Finding Study
Source: Interact J Med Res. 2015 Jan 13;4(1):e2. doi: 10.2196/ijmr.4022 (PMC4319080; doi:10.2196/ijmr.4022)
Supplement: Supplementary file 9 [file ijmr_v4i1e2_app9.pdf]

Multimedia Appendix 9. Unsupervised clustering of high risk population using PCA.

To reduce high dimensional EMR features for detecting cohort pattern, we used principle component analysis (PCA) to divide the high risk patients of future 6-month ED visit identified by our algorithm in the prospective cohort into distinctive groups, based on demographics, primary diagnosis and procedure, and chronic disease conditions. The features for high-risk patients are projected to a lower dimensional subspace with largest variances.

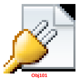

Where X_i is EMR feature matrix for each high-risk patient, and w_k is the set of vectors of weights that map each patient feature vector X_i to a new vector of principal component scores T_i^k. And we computed w_1 by solving following objective functions (1) and (2) and w_k by iterating objective function (3) based on the first k-1 principal components,

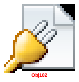

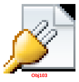

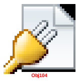

And then K-means algorithm was applied on the top of principal components T_i^k subspace of PCA to find potential patient patterns for future 6-month ED visit. We used K=6 to implement initial k means set for the algorithm and calculate the Euclidean centroid m to generate finial clusters,

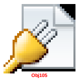

Where C_i is the i_{th} cluster in total 6 clusters, and x represents the previous principal components T^k.

Unique patterns revealed by the clustering results were analyzed to characterize the high-risk subjects identified by our ED algorithm. Unique patterns revealed by the clustering results were analyzed to characterize the high-risk subjects identified by our ED algorithm.
