# Supplementary material for: Real-Time Web-Based Assessment of Total Population Risk of Future Emergency Department Utilization: Statewide Prospective Active Case Finding Study
Source: Interact J Med Res. 2015 Jan 13;4(1):e2. doi: 10.2196/ijmr.4022 (PMC4319080; doi:10.2196/ijmr.4022)
Supplement: Supplementary file 12 [file ijmr_v4i1e2_app12.pdf]

Multimedia Appendix 12. Clustering of ED-6-month high-risk patients in the retrospective cohort according to demographics and the prior year clinical histories.

Categories	Characteristics	Cluster					
		1	2	3	4	5	6
Patient	Number	1439	1115	842	790	608	880
Resource consumption	Number of distinct chronic diagnoses - Mean	2.6	1.9	2.9	2.8	1.9	8.2
	Number Of laboratory test results - Mean	191	183.1	1	254.2	0.5	737.8
	Number Of radiographic tests results - Mean	3.2	2.8	2.1	3.9	1.1	7.9
Age groups	Age (0) - %	0.1	1.4	0.6	0.5	6.7	0
	Age (1-5) - %	0.6	1.7	3.3	1.4	6.6	0.3
	Age (6-12) -%	0.4	0.8	1.7	0.5	4.3	0.2
	Age (13-18) -%	2	2.6	3.7	3	3.1	0.5
	Age (19-34) -%	38.2	52.7	32.3	45.1	46.6	15
	Age (35-49) - %	29.1	22.9	26.6	23.7	17.9	25.8
	Age (50-65) - %	16.7	11.1	16.3	14.4	8.6	31.9
	Age (65+) - %	12.9	6.7	15.6	11.4	6.3	26.3
Gender	Male - %	35.1	41.8	39.2	43.2	51.3	36.1
Chronic diagnosis	With any chronic diagnosis - %	76.8	68.4	68.5	76.5	69.7	93.9
Chronic conditions	Endocrine, nutritional, and metabolic diseases and immunity disorders	31.7	20.5	20.9	29.8	16	71.9
	Diseases of the circulatory system	26.1	17.8	17.7	28.5	10.9	66.6
	Diseases of the nervous system and sense organs	35.5	27.4	27.6	31.4	19.6	61
	Diseases of the digestive system	20.9	15.7	14.1	22.7	9.2	55.3
	Diseases of the respiratory system	25.4	21.4	21.5	23.5	21.6	49.8
	Factors influencing health status and contact with health ser vices	7.6	5.4	4.9	9.1	3	34.6
	Diseases of the musculoskeletal system	18.4	10.7	14.4	13	7.7	38.4
	Diseases of the genitourinary system	15.1	11.8	8.9	17.2	4.9	30.8
	Diseases of blood and blood forming organs	4	3.5	2.4	6.3	1.3	19.7
	Symptoms, signs, and ill defined conditions	3.7	2.2	3.1	3	1.6	14.1

	Diseases of the skin and subcutaneous tissue	2	0.9	2.9	3	1.6	9.3
	Neoplasms	3.2	1.7	1.2	2.4	1.2	9.2
	Congenital anomalies	2.7	1.3	1.8	2.8	1.8	6.7
	Infectious and parasitic disease	2	1.4	0.8	1.1	0.7	3.5
	Injury and poisoning	0.3	0.3	0.2	0.5	0	2.2
	Complications of pregnancy, childbirth, and the puerperium	1.5	1.4	0.1	0.4	0	0.6
	Certain conditions originating in the perinatal period	0	0.1	0	0.1	0	0.1

Prospective clustering analysis of ED-6-month high-risk patients according to demographics and the prior year clinical histories

Categories	Characteristics	Cluster					
		1	2	3	4	5	6
Patient	Number	1236	1028	908	771	722	795
Resource consumption	Number of distinct chronic diagnoses - Mean	2.9	1.9	2.7	3.4	1.7	8.5
	Number Of laboratory test results - Mean	162.6	159.3	0.6	281	0.4	782.4
	Number Of radiographic tests results - Mean	3.3	3	2	4.4	1.2	8.5
Age groups	Age (0) - %	0.1	0.6	0.7	0.4	1.5	0
	Age (1-5) - %	0.7	2.6	4.2	2.1	9.8	0.1
	Age (6-12) -%	0.2	1.5	2.6	1	4.4	0.1
	Age (13-18) -%	2	3.8	4	3.9	5.7	0.5
	Age (19-34) -%	35	48.7	26.4	40.6	40.2	15
	Age (35-49) - %	30.8	23	26.3	22.2	21.1	25.8
	Age (50-65) - %	18.5	13.6	18.9	18.4	11.6	33.7
	Age (65+) - %	12.7	6.2	16.9	11.4	5.7	24.8
Gender	Male - %	36.1	41.4	41.9	43.3	52.6	40.1
Chronic diagnosis	With any chronic diagnosis - %	84.1	76.5	80.5	85.1	85.6	96.9
Chronic conditions	Endocrine, nutritional, and metabolic diseases and immunity disorders	36.9	23.8	31.8	36.6	19.8	74.2
	Diseases of the circulatory system	30	19.9	29.2	32.9	13.7	68.4
	Diseases of the nervous system and sense organs	38.7	28.9	32.4	33.1	26	66.5
	Diseases of the digestive system	24.3	17.4	18.5	25.9	14.5	55
	Diseases of the respiratory system	28.8	23.4	28.5	29.6	33.4	50.6
	Factors influencing health status and contact with health services	10	4.7	7.8	11.8	5	37
	Diseases of the musculoskeletal system	20.4	10.9	17.1	14.1	10.7	35.7
	Diseases of the genitourinary system	14.2	12.1	11.3	19.5	7.8	32.7
	Diseases of blood and blood forming organs	3.4	3	3.5	8.6	1.5	20
	Symptoms, signs, and ill defined conditions	4.9	2.4	2.9	4.3	0.8	15.1
	Diseases of the skin and subcutaneous tissue	1.6	1.9	3.7	3.4	1.4	8.6

	Neoplasms	3.1	1.5	2.3	4.2	0.6	8.4
	Congenital anomalies	2.4	1.8	2.2	3.9	2.8	5.5
	Infectious and parasitic disease	2.6	1.1	1.4	1.4	0.6	3.7
	Injury and poisoning	0.1	0.2	0.1	0.8	0	2.3
	Complications of pregnancy, childbirth, and the puerperium	1.5	1.1	0.2	1.6	1.1	0.6
	Certain conditions originating in the perinatal period	0	0	0	0	0.1	0
